# Bioadhesive Polymeric Films Based on Red Onion Skins Extract for Wound Treatment: An Innovative and Eco-Friendly Formulation

**DOI:** 10.3390/molecules25020318

**Published:** 2020-01-13

**Authors:** Cinzia Pagano, Maura Marinozzi, Claudio Baiocchi, Tommaso Beccari, Paola Calarco, Maria Rachele Ceccarini, Michela Chielli, Ciriana Orabona, Elena Orecchini, Roberta Ortenzi, Maurizio Ricci, Stefania Scuota, Maria Cristina Tiralti, Luana Perioli

**Affiliations:** 1 Department of Pharmaceutical Sciences, University of Perugia, via del Liceo 1, 06123 Perugia, Italy; cinzia.pagano@unipg.it (C.P.); maura.marinozzi@unipg.it (M.M.); tommaso.beccari@unipg.it (T.B.); paola.calarco@unipg.it (P.C.); chele@hotmail.it (M.R.C.); michela.chielli@virgilio.it (M.C.); maurizio.ricci@unipg.it (M.R.); maria.tiralti@unipg.it (M.C.T.); 2 Department of Molecular Biotechnology and Health Sciences, Sect. Analytical Chemistry, Via Pietro Giuria 5, 10125 Torino, Italy; claudio.baiocchi@unito.it; 3 Department of Experimental Medicine, Sect. Pharmacology, University of Perugia, P.le L. Severi 1, Bld C/4th floor, 06132 Perugia, Italy; ciriana.orabona@unipg.it (C.O.); elena.orecchini@gmail.com (E.O.); 4 Istituto Zooprofilattico dell’Umbria e delle Marche, via G. Salvemini, 1, 06126 Perugia, Italy; r.ortenzi@izsum.it (R.O.); s.scuota@izsum.it (S.S.)

**Keywords:** onion skins extract, hydrogel, polymeric films, anti-inflammatory, antibacterial

## Abstract

The onion non-edible outside layers represent a widely available waste material deriving from its processing and consumption. As onion is a vegetable showing many beneficial properties for human health, a study aiming to evaluate the use of extract deriving from the non-edible outside layers was planned. An eco-friendly extraction method was optimized using a hydroalcoholic solution as solvent. The obtained extract was deeply characterized by in vitro methods and then formulated in autoadhesive, biocompatible and pain-free hydrogel polymeric films. The extract, very soluble in water, showed antioxidant, radical scavenging, antibacterial and anti-inflammatory activities, suggesting a potential dermal application for wounds treatment. In vitro studies showed a sustained release of the extract from the hydrogel polymeric film suitable to reach concentrations necessary for both antibacterial and anti-inflammatory activities. Test performed on human keratinocytes showed that the formulation is safe suggesting that the projected formulation could be a valuable tool for wound treatment.

## 1. Introduction

The common onion (*Allium cepa* L.) is a worldwide cultivated vegetable used both as food and also in the health field for the presence of bioactive molecules mainly endowed with antioxidant activity [1,2,3]. Many scientific studies on the lipid- and glucose-lowering [4,5,6,7,8], anti-inflammatory [9,10,11], antioxidant [12,13,14,15,16] and antibacterial [13] properties of onion extracts have been published. Our previous work in this area reported the pungency determination [17] and the antioxidant and immune modulatory activities of purple-skin Rojo Duro onion extracts [18].

The non-edible outside layers (hereinafter referred as OL) are the main waste material obtained from onion processing and consumption. Besides rich in flavonoids [19], they are odorless and can be stored for a long time after the harvesting. The utilization of a biomass endowed with these positive peculiarities fulfills the principles of circular economy and meets the need of environmentally aware consumers [20].

The above considerations, along with the availability of onions endowed with short and traceable production-chain, pushed us to develop an innovative study based on OL use as source of active ingredients [21,22,23,24,25] for the preparation of dermatological products for wound treatment. The presented approach is advantageous as it proposes a new formulation by using waste materials reducing the disposal costs and the consequent environmental impact.

The work was structured as follows: (i) optimization of the extraction method according to green chemistry principles, (ii) evaluation of the extract activity in terms of antioxidant, radical scavenging, antibacterial, anti-inflammatory activities and cytotoxicity and (iii) formulation of the extract as an hydrogel film followed by its activity and safety evaluation.

## 2. Results

### 2.1. Optimization of the Extraction Procedure

The first step of the study was the set-up of the best extraction conditions in terms of recovery material, total phenol content (TPC) and antioxidant activity. On the road of biocompatibility, ultrapure water and EtOH were chosen as unique extraction solvents and the procedure was tested both at RT and 60 °C. The obtained results (Table 1) showed that increasing the temperature the recovery yield increased using both abs and 70% EtOH (Table 1); the presence of water in the extraction medium gave a higher extract recovery. 

**Figure 1:**
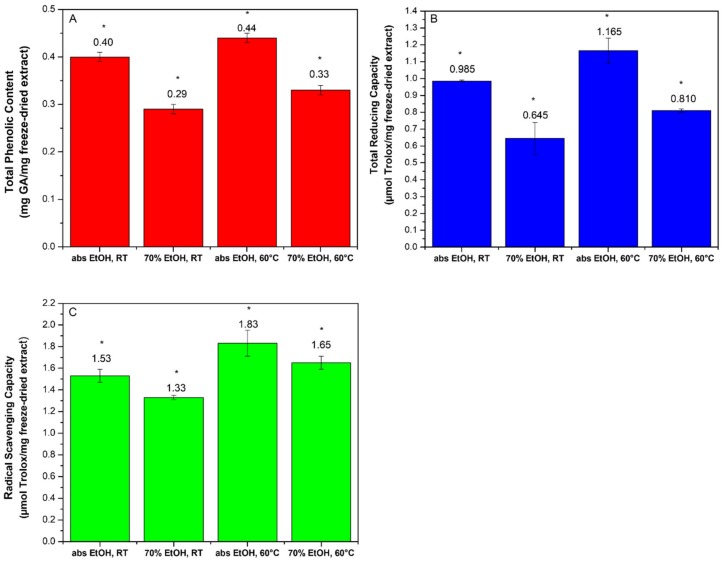
(**A**) Total phenol content (TPC) of the extracts obtained by the four different extraction conditions. Data are expressed as mg of GA/mg of freeze-dried extract and represent the mean of six samples, each measured in triplicate; * *p* ≤ 0.05 (one-way ANOVA test). (**B**) Total reducing capacity (TRC) of the extracts obtained by the four different extraction conditions. Data are expressed as mg of GA/mg of freeze-dried extract and represent the mean of six samples, each measured in triplicate. * *p* ≤ 0.05. (**C**) Radical scavenging capacity (RSC) of the extracts obtained by the four different extraction conditions. Data are expressed as mg of GA/mg of freeze-dried extract and represent the mean of six samples, each measured in triplicate; * *p* ≤ 0.05 (one-way ANOVA test).

TPC of the extracts obtained by the four different procedures was measured by the Folin–Ciocalteu method using a calibration curve of gallic acid standard solutions [18]. The calibration curve was fully validated for intra-day and inter-day precision and accuracy before the use (r = 0.9972). A statistically significant difference in TPC was observed among the samples resulting from the different extraction conditions: the use of abs EtOH increased the TPC of the corresponding extracts at both temperatures (Figure 1A). Although abs EtOH resulted a less efficient extraction solvent in terms of yield, it gave extracts with higher TPC than those obtained using 70% EtOH. Among the several protocols reported for assessing the free radical-trapping capability of phytochemicals [26], the ferric ion reducing antioxidant power (FRAP) and 2,2-diphenyl -1-picrylhydrazyl (DPPH) radical assays were chosen [27]. According to FRAP test results (Figure 1B) the highest total reducing capacity (TRC) was found in the extract obtained at 60 °C using abs EtOH. The data ranking of the four extracts was perfectly in accordance with TPC results, hypothesizing that TRC is attributable to the polyphenolic component. Again, the extract obtained in abs EtOH at 60 °C was endowed with the best performance also in DPPH assay (Figure 1C) [18]. The three assays therefore confirmed that the antioxidant component was more soluble in abs EtOH than in hydroalcoholic medium (EtOH 70%) and stable up to 60 °C. Due to the coherence of the obtained data, the extract obtained by using abs EtOH at 60 °C (hereinafter referred as OLE, outside layers extract) was selected for the further studies.

### 2.2. Extract Characterization

#### 2.2.1. Fingerprint Analysis of OLE Constituents

**Figure 2:**
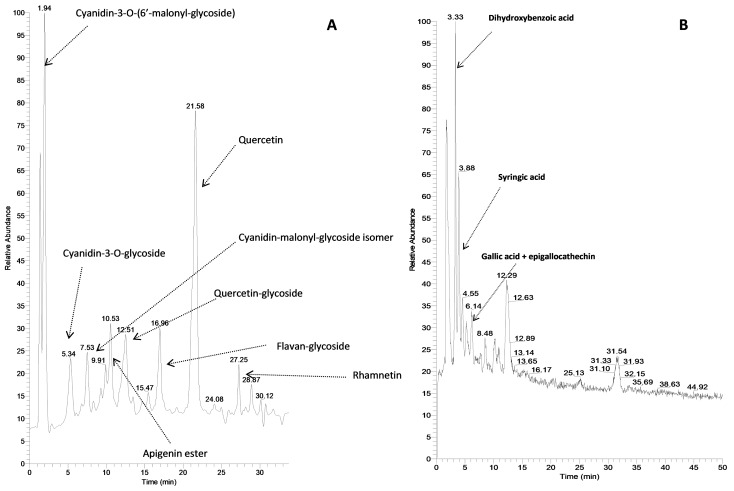
(**A**) Chromatographic separation of OLE constituents monitored in full mass ion positive mode and (**B**) chromatographic separation of OLE constituents monitored in full mass ion negative mode.

The main OLE components were identified. The species more present were the anthocyanidine cyanidin-3-O-(6′-malonyl-glycoside) (protonated precursor ion *m*/*z* = 535.1082) and the flavonol quercetin (protonated precursor ion *m*/*z* = 303.0504). Two other anthocyanidines were present (responsible together with the previously cited one of the red color of the onion) positional isomers of cyanidin malonyl glycoside (*m*/*z* = 535.1082) and a cyanidin glycoside (*m*/*z* = 449.1081). Quercetin is only present in glycosylated form (*m*/*z* = 465.1028) and in much less amount in diglycosilated form (*m*/*z* = 627.1881). In Figure 2A a chromatogram of OLE monitored in full mass is reported showing the main components identified. The UV spectra (not reported) were highly helpful in substances identification because their shape were informative about the chemical class the various molecules belonged to. Figure 2B shows the separation of much more polar substances performed in isocratic conditions and monitored in ion negative mode. It can be detected that they show very short retention times. Epigallocatechin has anti-oxidative properties whereas the other three acidic substances possess anti-bacterial properties. A quantitative analysis for both cyanidin derivatives and quercetin was performed using quercetin and cyanidin pure standards. The obtained results were: quercetin 9.5 ppm, cyanidin-3-O(-6′-malonyl-glycoside) 5.2 ppm, cyanidin malonyl glycoside isomer 1.7 ppm and cyanidin glycoside 2.1 ppm.

All these substances and the other flavonoids present in smaller amounts exhibit anti-oxidative activities combined with the anti-bacterial properties of benzoic acid derivatives.

#### 2.2.2. Antibacterial Activity Assay 

Preliminary experiments demonstrated that *Staphylococcus epidermidis*, *Staphylococcus aureus*, *Listeria innocua* and *Enterococcus faecalis* are sensitive to OLE, for this reason Minimum inhibitory (MIC) and minimum bactericidal (MBC) values were measured. The obtained results show the lowest MIC and MBC values for both *S. epidermidis* and *S. aureus* therefore the most sensitive to OLE among the investigated strains (Table 2). The values measured for *S. epidermidis* are the closest to the positive control ampicillin suggesting its greater susceptibility to OLE in comparison to the other ones. 

#### 2.2.3. Cytotoxicity Studies on RAW 264.7 and HaCaT Cell Lines

**Figure 3:**
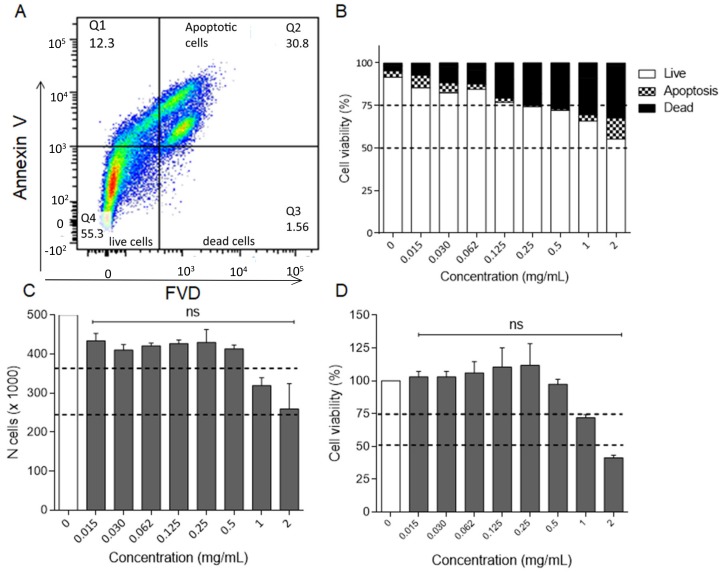
(**A**) LPS-activated cells were treated for 24 h with OLE at the indicated concentrations. Cells were stained using the PerCP-Annexin V and FVD 780 and analyzed by flow cytometry. Annexin V/FVD—double negative cells (lower left quadrant) represented live cells, annexin V/FVD—double positive cells (upper right quadrant) represented apoptotic cells and annexin V-negative/FVD-positive cells (lower right quadrant) indicated dead cells. A representative dot plot is shown. The percentage of viable, apoptotic and dead cells was reported in (**B**) for each OLE concentration. Data are the mean percentage of two different experiments. Evaluation of OLE cytotoxicity and safety on HaCaT cell line by (**C**) Trypan Blue exclusion and (**D**) MTT assays. Dotted lines indicate the 50% and 75% of cell viability. ns, not significant OLE-treated versus untreated group (one-way ANOVA test).

As macrophages play a key role in all phases of wound healing (inflammation, proliferation and remodeling) [28], RAW 264.7 cell model was used to evaluate OLE anti-inflammatory activity. Firstly, in order to exclude false positives, OLE cytotoxicity on the macrophage cell line RAW 264.7 stimulated with lipopolysaccharide (LPS, 50 ng/mL for 24 h) was investigated. By using eight two-fold dilutions of OLE in the 0.015–2.0 mg/mL concentration range, after 24 h of incubation, it was observed that the cell viability decreased below 75% at the concentration of 0.5 mg/mL. However, it was maintained higher than 65% up to 1 mg/mL and dropped around 50%, with an increased fraction of dead/apoptotic cells, at the concentration of 2.0 mg/mL (Figure 3A,B). For this reason, the concentration 2 mg/mL was excluded from the study focusing the attention on the concentrations range of 0.015–1.0 mg/mL for testing a potential OLE anti-inflammatory effect in LPS-treated RAW 264.7 cells.

Simultaneously, Trypan Blue exclusion assay and MTT test were carried out on human immortalized keratinocyte cell line (HaCaT), in vitro model of stratum corneum, to evaluate OLE safety in the same concentration range used for RAW 264.7 cells (0.015–2.0 mg/mL). The dose-response curve obtained from Trypan Blue exclusion assay revealed that the number of cells was comparable to the negative control until 0.5 mg/mL and slightly decreased at 1 mg/mL, reaching the 50% of viability at 2.0 mg/mL (Figure 3C). Similar results were obtained by MTT assay. By using the same OLE concentration range (0.015–2.0 mg/mL), after 24 h of incubation HaCaT cells viability was around 75% up to 1 mg/mL, decreasing below 50% at 2.0 mg/mL (Figure 3D). Overall, the cytotoxicity analyses revealed that OLE is safe for both cell lines in the concentration range 0.015–0.5 mg/mL. 

#### 2.2.4. Anti-Inflammatory Activity

**Figure 4:**
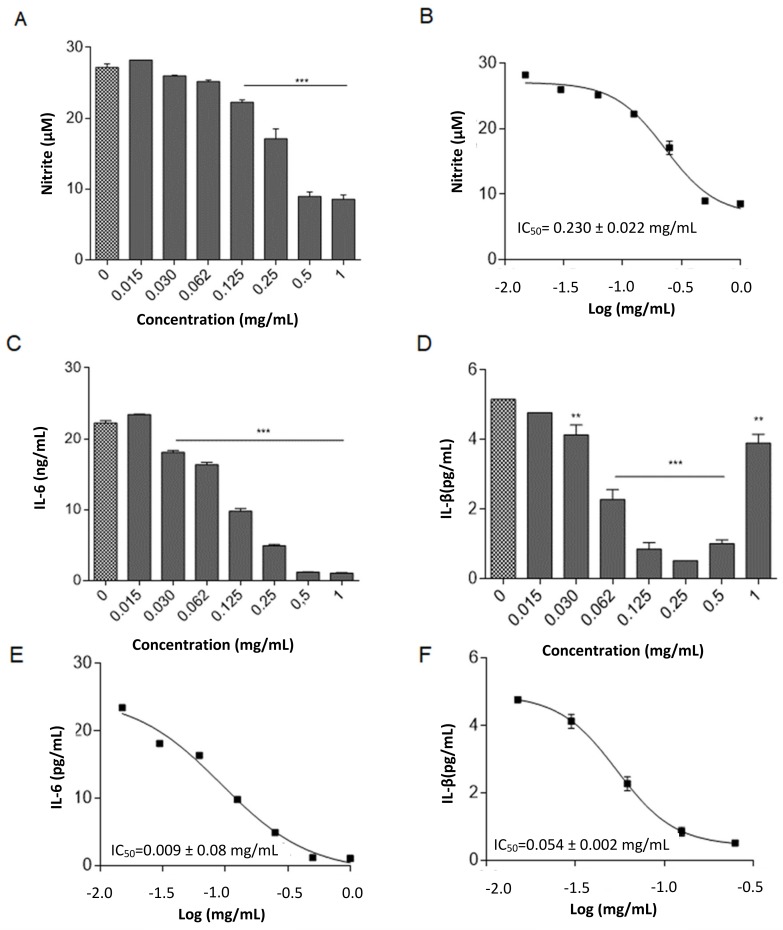
(**A**) LPS-activated RAW 264.7 cells were in vitro stimulated using different OLE concentrations for 24 h. NO release in the supernatant culture was quantified by using Griess reagent. Results are reported as mean ± SD of three independent experiments, each conducted in triplicate. (**B**) *** *p* < 0.0001, OLE–treated versus LPS-treated group (one-way ANOVA test). Concentration-response curve was obtained for the determination of the IC_50_. Results are reported as mean of two independent experiments, each conducted in triplicate. LPS-activated RAW 264.7 cells were in vitro stimulated with different concentrations of OLE for 24 h. Supernatants were collected and the concentrations of IL-6 and IL-1β were determined by ELISA test (**C**,**D**). ** *p* < 0.001, *** *p* < 0.0001, OLE-treated versus LPS-treated group (one-way ANOVA test). Concentration-response curves were obtained for the determination of the IC_50_. For each curve results are reported as mean of two independent experiments, each conducted in triplicate (**E**,**F**).

RAW 264.7 stimulation by LPS for 24 h induces NO synthase (iNOS) activation and thus NO release in the culture supernatant [29], detectable by using Griess reaction. The incubation of LPS stimulated RAW 264.7 with OLE for 24 h induces a significant decrease of NO release in the concentration range 0.125–1.0 mg/mL (Figure 4A). The obtained concentration curve provided an IC_50_ = 0.230 ± 0.022 mg/mL for the down-regulation of NO release by OLE (Figure 4B). The release of pro-inflammatory cytokines, IL-6 and IL-1β in the same cell system was analyzed as reported in the literature [30,31]. It was observed a similar inhibitory effect exerted by OLE on the production of both cytokines in LPS-treated RAW 264.7 macrophages. Specifically, both IL-6 and IL-1β were significantly inhibited by OLE starting from the concentration of 0.030 mg/mL (Figure 4C,D). The inhibitory effect on IL-6 production was concentration-dependent, with an IC_50_ = 0.090 ± 0.008 mg/mL (Figure 4E). Differently, the inhibitory effect on IL-1β production showed a concentration-dependence in the range 0.030–0.25 mg/mL, with an IC_50_ = 0.054 ± 0.002 mg/mL (Figure 4F). For higher concentrations (0.5–1.0 mg/mL) a decreased inhibitory effect on IL-1β production was observed, probably due to the activation of the inflammasome in increased apoptotic RAW 264.7 cells [32,33].

Overall, OLE inhibited the production of inflammatory mediators in LPS-treated RAW264.7 cell line with IC_50_ lower than the highest cytotoxic concentration (i.e., 0.5 mg/mL).

### 2.3. Hydrogel Film Preparation

After these preliminary studies on pure OLE, a suitable dosage form was developed and characterized. The antioxidant, anti-inflammatory and antibacterial activities observed for pure OLE suggested that it could be a suitable active ingredient for wounds treatment. The wound healing process is a natural post-trauma repairing course rather complex and sensitive; its interruption can lead to the formation of non-healing chronic wounds. A chronic wound, in fact, can be defined as a lesion in which the normal healing process has been interrupted at one or more points during the phases of hemostasis, inflammation, proliferation and remodeling of the wound [34]. Diabetes, venous or arterial disease, infections, metabolic deficiencies, oxidative phenomena and inflammation are the main factors contributing to non-healing chronic wounds [35]. 

In order to exploit all the OLE biological activities a formulation suitable to be applied on severely injured skin was developed. This formulation should (i) auto-adhere to skin (without the aid of glue), (ii) protect the wound from mechanical solicitations, (iii) avoid occlusion and pain and (iv) be easily removable and able to promote a sustained OLE release.

In order to reach this objective, OLE was formulated in a hydrogel film for potential dermal applications. The formulation was projected and developed based on the following requirements: (i) biocompatibility, (ii) autoadhesivity to skin/wound, (iii) easy and pain-free removal (atraumatic removal) and (iv) an easy and scalable manufacturing method. The hydrogel films were prepared starting from hydrogels whose composition was optimized referring to a previous study [36] by using NaCMC (2%) and PVP K90 (0.1%) as bioadhesive polymers [37,38]. Many modifications in the composition were tested in order to find the most suitable for the hydrogel film preparation (Table S1). Differently from the previously developed films [36], bentonite nanoclay was introduced in the composition as filler [39] to improve the hydrogel film mechanical properties. A preliminary selection was made based on the following criteria: (i) hydrogel aspect (homogeneity and consistency) and physical stability, (ii) easy casting (difficult for very viscous gels) and (iii) final film appearance (detection of visible imperfection under visual inspection). Hydrogel 1 showed low consistency and instability due to bentonite particles sedimentation after 24 h from the preparation.

This phenomenon was ascribed to the low hydrogel viscosity and for this reason three new hydrogels (2, 3 and 4) were prepared with an increased amount of bentonite (Table S1) well known as rheological modifier agent [40]. The idea was that the increase of bentonite content could improve the hydrogel viscosity and thus its stability. Since, the sedimentation phenomenon was still observed, the amount of bentonite was fixed at 4.0% and NaCMC content increased until 3.0% (hydrogel 5, Table S1). These modifications allowed us to obtain a stable hydrogel, but very viscous and thick making difficult the successive casting procedure. To solve this problem hydrogel 6 was then prepared, having the same composition of hydrogel 1, with an increased NaCMC content (3.0%). The obtained hydrogel showed stability and suitable consistency to allow an easy casting. 

### 2.4. Hydrogel Film Characterization

#### 2.4.1. Hydrogel Film Antibacterial Activity 

Starting from hydrogel 6 composition (Table S1), three different hydrogel films were prepared loaded with 1.0, 3.0 or 5.0% w/w of OLE (Table S2). The corresponding hydrogel films were submitted to antibacterial activity studies in order to evaluate their ability to reach OLE concentrations necessary active against bacteria. Thus, the hydrogel films were evaluated against the same bacterial strains resulted sensitive to the unformulated OLE: *S. epidermidis, S. aureus, L. innocua* and *E. faecalis.* Inhibition halos of different sizes were generated by the three different films on the analyzed bacterial strains as reported in Table 3. Specifically, the hydrogel film B1 produced inhibition zone just for *S. epidermidis* and *S. aureus*, while B2 and B3 hydrogel films, containing OLE in higher amount, produced similar inhibition halos for all the tested bacterial strains (Table 3). Analogously to the results obtained for the unformulated OLE, *S. epidermidis* resulted in the most sensitive strain to the loaded hydrogel film. Overall, these data suggested that the hydrogel film B2, containing 10.92 mg/cm^2^ of OLE, could be the most suitable formulation for wounds application. For this reason further characterized in the next studies.

The preliminary thermal characterization of the hydrogel film B2 showed a water content of 13.6% (Figure S1) after drying and a high glass transition temperature (>200 °C; Figure S2). 

#### 2.4.2. Hydrogel Film Thickness, Swelling Behavior and Matrix Erosion Capacity

The hydrogel film B2 (circle 3.14 cm^2^) in dry conditions showed a thickness of 0.43 mm (±0.05), resulting very thin. In general, the low thickness of a film for skin use represents a suitable property for ensuring imperceptibility after the application. After hydration the film thickness increased to 2.5 mm (±0.04), suggesting that its swelling after the contact with SWF should not cause an excessive increase of the dimensions that could compromise patient’s acceptability during the use. The swelling ability of the hydrogel film B2 was an important parameter to be evaluated as this property is responsible for the ability to absorb exudate from the wound. Moreover, it influences the bioadhesion capacity and OLE release rate from the formulation.

The hydrogel film B2 exhibited a high capacity to absorb fluids, testified by the swelling % measured. It swelled reaching two fold its original weight after 15 min and nine fold after 8 h (Figure S3). This behavior can be attributed to the hydrophilic properties of the hydrogel film with a marked water affinity. During the experiment the hydrogel film showed also weight loss, expressed as erosion matrix % (Figure S3) and due to the gradual dissolution in the medium. The erosion % was 40% after 15 min, then reached 60% after 60 min, maintaining this extent until the 8th h. Both swelling and erosion % cannot be measured after 8 h as after this time the polymeric network resulted completely relaxed due to the very high amount of the absorbed SWF. Thus, the complete hydrogel film dissolution in SWF was observed. Based on these results, it is reasonable to think that the hydrogel film can be easily removed by washing ensuring an atraumatic and pain free removal.

#### 2.4.3. Ex Vivo Adhesion Studies 

**Figure 5:**
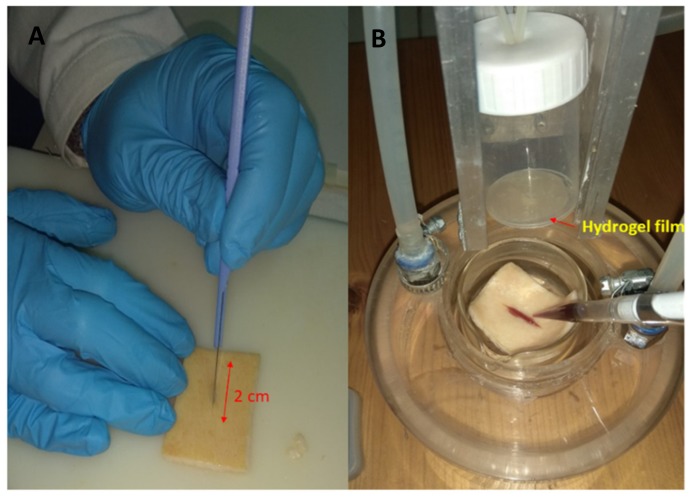
Skin used for the assay (**A**) and simulation of an open wound (**B**).

The adhesion capability of the hydrogel film B2 was evaluated ex vivo by using pig skin samples in which a wound was simulated as reported in Figure 5. The obtained results showed a detachment force of 0.4 N ± 0.06 and detachment time of 13.00 sec ± 0.57. The measured adhesion capacity of the hydrogel film B2 can be ascribed to the combination of both hydrophobic and hydrophilic interactions. The hydrophobic interactions predominate in the case of the binding between hydrogel film and stratum corneum of the peri-wound area. The hydrophilic interactions become more important in the binding of the damaged area characterized by the presence of exudate.

The obtained results suggested that the developed hydrogel film B2 possesses the suitable balance of hydrophobic and hydrophilic groups to bind wounded skin. In fact, the high swelling capacity measured (Section 3.5.2) evidenced that it is able to interact with the exudate and to swell. This event allowed the distension of NaCMC and PVP K90 polymeric chains exposing the hydrophilic groups (-OH and carboxyl groups for NaCMC and carbonyl for PVP K90) to establish interactions (mainly hydrogen bonds) with the subcutaneous tissues surrounding the wound.

The binding to the peri-wound area could mainly be attributed to hydrophobic groups (-CH_3_ for NaCMC and alkyl group for PVP K90) exposed to the outer side of the film and thus available to interact with skin. This aspect is very important as the hydrogel film composition is suitable to adhere to the skin surface avoiding the use of adhesives, not recommended in the open wounds management because they are painful and discomfortable.

#### 2.4.4. OLE In Vitro Release and Correlation with the Anti-Inflammatory Activity

**Figure 6:**
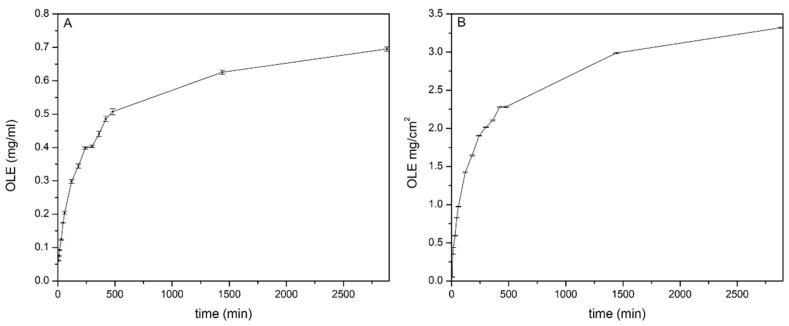
(**A**) OLE In vitro release profile of from the hydrogel film B2 represented as mg/mL vs. time and (**B**) mg/cm^2^ vs. time.

The release capability of hydrogel film B2 loaded with OLE was evaluated in vitro by the Franz diffusion cell. The obtained profile (Figure 6A) shows that OLE is released just after application reaching a concentration of 0.060 mg/mL in 15 min and 0.62 mg/mL within 24 h. This suggests that the hydrogel film B2 is able to release an amount of OLE necessary to obtain the anti-inflammatory activity (Section 2.2.4) from the first minutes. At the same time OLE level remained below 1.0 mg/mL, a viable concentration for both RAW 264.7 and HaCaT cell lines (Figure 3).

Despite the hydrogel film B2 has been projected for one daily application, OLE release monitoring was performed until 48th h in order to evaluate if the produced concentrations remain in the safety range. As reported in Figure 6A the amount of OLE released within 48 h increased slightly (0.69 mg/mL) compared to 24th h. The concentration remained below the cytotoxic value (1.0 mg/mL) suggesting that a prolonged application time did not impair skin cells viability.

It is interesting to evaluate the amount of OLE released per unit area (mg/cm^2^) as reported in Figure 6B. The hydrogel film B2 was able to produce effective concentrations per cm^2^ for the antibacterial and anti-inflammatory activities. Thus, wounds of different sizes could be treated modulating the hydrogel film and is a suitable delivery system for OLE dermal applications for wounds treatment.

#### 2.4.5. In Vitro Safety Studies of Hydrogel Film on HaCaT Cell Line

**Figure 7:**
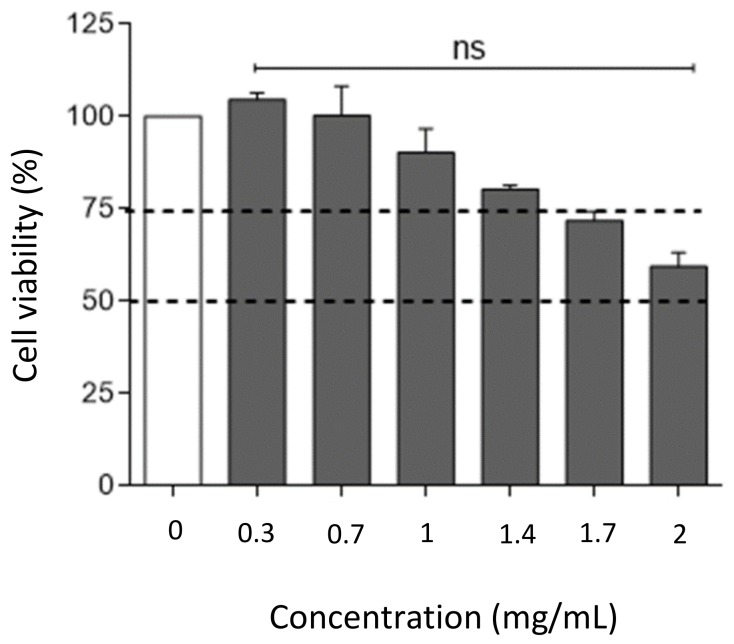
Evaluation of hydrogel film B2 cytotoxicity and safety on HaCaT cell line by an MTT assay. Dotted lines indicate the 50% and 75% of cell viability. ns, not significant OLE-treated versus untreated group (one-way ANOVA test).

As the hydrogel film was projected for one daily application the safety studies on HaCaT cells was evaluated within 24 h. A circular film (3.14 cm^2^) was incubated for 24 h at 37 °C in DMEM complete medium. Subsequently, the medium was used to treat HaCaT cells for 24 h. From the in vitro release studies it was possible to know the exact OLE amount released from the hydrogel film B2. Different concentrations comparable to OLE values (0.25, 0.5, 1, 1.5 and 2.0 mg/mL) were assayed. The obtained results from MTT assay showed very similar results compared to unformulated OLE (Figure 7) suggesting that both OLE alone and formulated in the hydrogel film B2 is cytotoxic at the concentration of 2.0 mg/mL (above the maximum concentration obtained from the hydrogel film). Thus, it is possible to conclude that both OLE and hydrogel film B2 are safe on an in vitro skin model. 

## 3. Materials and Methods 

### 3.1. Materials

Rojo Duro onion samples were provided by the farm “Azienda Agraria Turrioni Fiorella” (Cannara-Perugia, Italy). The extract from OL was prepared about five months after the onion harvesting.

Folin–Ciocalteu reagent, 2,4,6-tris(2-pyridyl)-*s*-triazine (TPTZ), 6-hydroxy-2,5,7,8-tetramethyl-2-carboxylic acid (Trolox), 2,2-diphenyl-1-picrylhydrazyl (DPPH), hydrochloric acid (HCl), ferric chloride (FeCl_3_), sodium acetate (NaOAc), sodium carbonate (Na_2_CO_3_), acetic acid (AcOH), gallic acid (GA) and ethanol (EtOH) and bentonite nanoclay were purchased from Sigma-Aldrich (Milano, Italy). Polyvinylpyrrolidone K90 (PVP K90) was furnished by ISP (Baar, Switzerland). Sodium carboxymethylcellulose (NaCMC) was purchased from Caelo (Hilden, Germany). Ultrapure water was obtained from a reverse osmosis based Milli Q System (Millipore, Milano, Italy). Other reagent grade chemicals and solvents were used without further purification. Ultrapure water was sterilized in a steam autoclave (121 °C, 2118 millibar absolute pressure and relative pressure 1360 millibar). 

The simulated wound fluid (SWF) pH 6.5 was prepared by dissolving 8.30 g of NaCl and 0.28 g of CaCl_2_ in 1000 mL of ultrapure water [36]. 

### 3.2. Extraction Procedure

OL, usually produced during the manipulation and braiding of fresh onions, were collected, then quickly washed with ultrapure water and finally dried by a cotton towel. OL (4 g) were suspended in 160 mL of absolute (abs) or 70% EtOH and the obtained suspension kept under magnetic stirring for 90 min at room temperature (RT) or at 60 °C. The supernatant was recovered by decantation (or by the use of a Pasteur pipette) and the residue resuspended in the same starting solvent (100 mL), kept under magnetic stirring for 90 min at the same previously used temperature. In this case, the supernatant was also recovered and the second extraction step repeated once again. The collected supernatants were combined and the solvent removed by a rotary evaporator (water bath temperature 37.0 ± 0.1 °C, Buchi Italia s.r.l., Cornaredo, Italy). The obtained solid was suspended in ultrapure water (28 mL) and the resulting suspension centrifuged at 4000 rpm, 20 °C for 20 min. The supernatant solution was recovered and the pellet twice submitted to the centrifugation step using 10 mL and 6 mL of ultrapure water for suspending the second and the third pellet, respectively. The combined supernatants were freeze-dried and the obtained dry material stored at −20 °C. The procedures were performed in duplicate for each extraction method. 

Folin–Ciocalteu assay [18] was performed using the sample (extract) dissolved in ultrapure water, whereas in the case of FRAP (ferric ion reducing antioxidant power) and DPPH (2,2-diphenyl -1-picrylhydrazyl) assays [18], the sample was dissolved in EtOH 75%. In any case, the solution was prepared dissolving 20 mg of freeze-dried extract in 50 mL of solvent (0.4 mg/mL).

### 3.3. Extract Characterization

#### 3.3.1. Fingerprint Analysis of OLE Constituents

A qualitative evaluation of OLE constituents was performed by LC-UV-high resolution mass spectrometry. Freeze-dried OLE (100.0 mg) was solubilized in 10.0 mL of an EtOH/water (70:30) solution. The obtained solution was centrifuged and 20.0 µL of the clear supernatant were injected in a LC-UV-Vis-HRMS (High resolution mass spectrometry) system. The LC module was an Ultimate 3000 (Thermo Scientific, Rodano, Milan, Italy), the UV-Diode Array module was a Surveyor PDA Plus Detector (Thermo Scientific, Rodano, Milan, Italy), and High Resolution Mass Spectrometer was a linear ion trap linked to an Orbitrap System (LTQ XL, Thermo Scientific, Rodano, Milan, Italy).

OLE molecular species were separated by using a Luna Phenomenex RP-18 column (150 mm × 2.1 i.d., 3 µm particles). Mobile phase was a binary mixture of 0.01% formic acid aqueous solution (solvent **A**) and acetonitrile (solvent **B**) pumped through the column at a flow rate of 0.200 mL/min. Two different chromatographic conditions were used aimed to suitably separate components with very different polarity properties and mass spectrometric response. More polar compounds were separated in isocratic conditions (**A**/**B** = 72/28) whereas the medium polarity constituents were separated by a gradient elution program consisting of a linear gradient from **A**/**B** = 95/5 to **A**/**B** = 50/50. UV-Vis detector was programmed in the wavelength range 220–650 nm.

High resolution and LC-tandem mass spectra were acquired either in negative and in positive ion mode using a linear ion trap-Orbitrap detector equipped with an ESI source with the following parameter settings: spray voltage 4.5 kV; sheath gas flow rate 35 (arbitrary units); drying gas flow rate 25 (arbitrary units) and capillary temperature of 250 °C.

Full scan mass spectra were recorded in the range *m*/*z* 220–1200. MS/MS spectra were acquired in the range between ion trap cut-off and precursor ion m/z values. High resolution mass accuracy of recorded ions (vs. calculated) was ±5 millimass units (without internal calibration). High-resolution spectra were acquired with the resolution R = 30,000 (FWHM).

#### 3.3.2. Antibacterial Activity Assay

OLE antibacterial activity was evaluated for four bacterial species *S. epidermidis* WDCM (world data centre for microorganisms) 00036, *S. aureus* WDCM 00034, *E. L. innocua* WDCM 00017 and *faecalis* WDCM 00087. MIC and MBC concentrations were measured using a standard microdilution technique according to Clinical Laboratory Standards Institute Guidelines, adapting the protocol as suggested by J.M. Silván et al. [41]. The bacterial suspension used for the assay was prepared using bacteria to approximately 1 × 10^5^ CFU/mL in Muller Hilton Broth (MHB; Biolife Italiana s.r.l, Cod. 4017412). The stored strains were revitalized on Brain Heart Infusion Broth (BHI, Biolife Italiana s.r.l, Cod. 4012302) and incubated according to their own growth conditions (Table S3). At the time of use, OLE was dissolved in sterile ultrapure water to obtain the concentration required by the protocol for the evaluation of the antibacterial effect. The assayed concentrations were: 30, 15, 7.50, 3.75, 1.88, 0.94 and 0.47 mg/mL. Moreover, for each bacterial strain three controls were set up. These included antibiotic control (ampicillin), organism control (MHB and the bacterial suspension), negative control (MHB and the solution of the extract at the same concentration tested). For each bacterial species the test was performed in triplicate. The microplates were incubated according to the growth conditions of each bacterial strain tested (Table S3). After incubation, serial decimal dilutions of each well were prepared in sterile saline solution (0.9% *w*/*v* NaCl) and plated onto agar with 5% sheep blood (Biolife Italiana s.r.l, Cod. 4011552). Results are expressed as log CFU/mL.

#### 3.3.3. Cell Lines and Cytotoxicity Studies

The mouse macrophage cell line RAW 264.7, obtained from the American Type Culture Collection (ATCC, Manassas, VA, USA), was used to investigate OLE anti-inflammatory activity. RAW 264.7 cells were cultured according to standard procedures in Roswell Park Memorial Institute 1640 medium (RPMI-1640), whereas HaCaT cells were cultured according to standard procedures in Dulbecco’s modified Eagle’s medium (DMEM). Both medium were supplemented with 10% heat-inactivated Fetal Bovine Serum (FBS), 2 mM of L-glutamine and antibiotics (100 U/mL penicillin, 100 μg/mL streptomycin; Gibco, Invitrogen, Carlsbad, CA, USA). Both cell lines were cultured at 37.0 °C in a 5% CO_2_ atmosphere and the medium replaced every 3 days. RAW 264.7 and (HaCaT) were tested for mycoplasma contamination before use. RAW 264.7 cells were activated with lipopolysaccharides (LPS), serotype 055:B5 (Sigma-Aldrich, Saint Louis, MO, USA) at 50 ng/mL for 24 h. LPS-activated RAW 264.7 cells were cultured at the concentration of 1 × 10^6^ cells/700 μL in a 24-well plate and co-treated with serial two-fold dilutions of OLE for 24 h. The percentage of live, apoptotic and dead cells, was determined by using Annexin V Apoptosis Detection Kit PerCP-eFluor™ 710 and FVD (Fixable Viability Dye eFluor™ 780; eBioscience, San Diego, CA, USA), according to the manufacturer’s instructions. Specifically, each cell sample was washed and suspended in 100 µL of phosphate-buffered saline before the staining. A dilution 1:1000 of FVD was then added to the sample and incubated at 4 °C for 30 min in the dark. Annexin V-PerCP, diluted 1:20, was added and incubated for 15 min at RT in the dark. Flow cytometry analysis was performed within 4 h.

HaCaT cells were purchased from I.Z.S.L.E.R. (Istituto Zooprofilattico Sperimentale della Lombardia e dell’Emilia Romagna, Brescia, Italy) and used as model for assessing the epidermal homeostasis during wound treatment. Cells viability was assessed after treatment for 24 h with OLE both by Trypan Blue exclusion and MTT assays [42]. Cells were seeded at the density of 2 × 10^5^ cells/well into 6-well culture plates in a final volume of 2 mL and 5 × 10^3^ cells/well into 96-well flat bottom culture plates in a final volume of 200 μL, for Trypan Blue exclusion and MTT assay respectively. Cell viability was measured and expressed as a percentage relative to that of the control cells as previously described [43].

#### 3.3.4. Anti-Inflammatory Activity

For the quantification of nitric oxide (NO) release, LPS-activated RAW 264.7 cells were cultured at the concentration of 0.15 × 10^6^ cells/200 μL in a 96-well plate and co-treated with serial two-fold dilutions of OLE for 24 h. The total production of NO was detected by incubating at RT for 7 min each culture supernatant (50 μL) with 100 μL of Griess reagent (1% naphthylethylenediamine dihydrochloride in distilled water and 1% sulfanilamide in 5% concentrate H_3_PO_4_, mixed 1:1). Nitrite concentration was quantified by comparison with a sodium nitrite standard curve. The absorbance of samples was read at λ= 530.0 nm, by using a spectrophotometer (TECAN). The assay was conducted in triplicate.

For the quantification of IL-6 and IL-1β, LPS-activated RAW 264.7 cells were cultured at the concentration of 0.5 × 10^6^ cells/500 μL in a 48-well plate and co-treated with serial two-fold dilutions of OLE for 24 h. IL-6 and IL-1β were determined in the culture supernatants by Mouse IL-6 Uncoated ELISA kit (Invitrogen, Carlsbad, CA, USA) and Mouse IL-1 β ELISA Ready-SET-Go!™ kit (eBioscience, San Diego, CA, USA), respectively, as described by the manufacturer’s instructions. The experiments were conducted in triplicate and repeated two times. 

### 3.4. Hydrogel Film Preparation

The hydrogel films were prepared by the solvent casting method [36] starting from a hydrogel. An OLE solution (1.0%, 3.0% or 5.0% *w*/*w*) was initially prepared in ultrapure water then, bentonite (1%) was dispersed in this solution. Finally, glycerol (10%), NaCMC (3.0%) and PVP K90 (0.1%) were added. The mixing was performed at 600 rpm, for 20 min, at RT using a mechanical stirrer equipped with a three blade helical impellers (DLS VELP^®^ Scientifica, Usmate MB, Italy). In order to remove the air incorporated during the mixing, each hydrogel was degassed in a conditioning planetary mixer (Thinky mixer ARE-250) at 2000 rpm for 10 min. Afterwards, 3.5 g of the prepared mixture was casted into silicon molds (ø = 3.5 cm) and left to dry in ventilated oven at 37.0 °C ± 0.1 for 24 h. After drying, the hydrogel films were stored at RT and 40% relative humidity (RH) until use.

### 3.5. Hydrogel Film Characterization

#### 3.5.1. Hydrogel Film Antibacterial Activity

The test medium and bacterial suspension used for the assay were prepared as previously reported (Section 3.3.2). The experiment was carried out on *S. epidermidis* WDCM 00036, *S. aureus* WDCM 00034*, E. faecalis* WDCM 00087 and *L. innocua* WDCM 00017. Different inoculated media were used for each bacterial strains. Sterile Petri dishes (diameter 90 mm) were filled with 20 mL of the prepared and seeded media. After medium solidification, a small square (1 cm × 1 cm) of the hydrogel film was placed in each series of plates and incubated according to their own growth conditions (Table S3). The test was conducted in triplicate for each bacterial strain. Negative control was set up using a hydrogel film OLE free. At the end of the incubation time the presence and the diameter of the inhibition halo was evaluated by a gauge.

#### 3.5.2. Hydrogel Film Thickness, Swelling Behavior and Matrix Erosion

Hydrogel film thickness was measured in both dry and wet state by a manual micrometer (Borletti, Cremona, Italy). The thickness of the hydrogel film after hydration was measured after incubation in 10 mL of SWF thermostated at 32.0 ± 0.5 °C for 8 h. After this period, the excess of SWF was removed by filter paper and the thickness measured. The hydrogel film swelling capacity and erosion [44] were calculated using Equations (1) and (2) respectively:

(1) $$\mathrm{swelling}\;\%\;=\;\frac{W2-W1}{W1}\;\times \;100.$$

(2) $$\mathrm{erosion}\;\%\;=\;\frac{W1-W3}{W1}\;\times \;100.$$

Each hydrogel film (circles of 3.14 cm^2^) was weighted (W1), immersed in 10 mL of SWF into a Petri plate (ø = 5 cm) and thermostated at 32.0 ± 0.1°C for established times (15, 30, 60, 90, 120, 180, 300 and 1440 min). After immersion, the hydrogel films were wiped off from the excess of SWF using filter paper and weighted (W2).

The erosion % was measured as follows. The swollen films were dried at 60 °C for 24 h and kept in desiccator over CaCl_2_ (40% RH) for 48 h and after drying the weighting was repeated (W3). The obtained results represent the average of three measurements (*n* = 3).

#### 3.5.3. Thermal Properties Measurement

Thermogravimetric (TGA) analysis was performed by a Netzsch STA 449C apparatus (Mettler Toledo, Milano, Italy), in air flow and heating rate of 10 °C/min, to determine the weight loss as a function of increasing temperature. Differential scanning calorimetry (DSC) analysis was performed using an automatic thermal analyzer (DSC821e, Mettler Toledo, Milano, Italy) and indium standard for temperature calibrations. Holed aluminum pans were employed in the experiments for all samples and an empty pan, prepared in the same way, was used as a reference. Samples of 3–6 mg were weighted directly into aluminum pans and thermal analyses of the samples were conducted, at a heating rate of 5 °C/min from 25 to 300 °C.

#### 3.5.4. Ex Vivo Adhesion Studies

The hydrogel film adhesion force and time were assessed using pig skin samples (from shoulder region), obtained from large white pigs weighing 165–175 kg, furnished by the Veterinary Service of ASL N.1 Città di Castello (Perugia, Italy) and used within 12 h from pig death [36]. The ex vivo adhesion force was measured by a dynamometer (Didatronic, Terni, Italy). By means of cyanoacrylate glue the film was attached to the bottom of a cylindrical support and the porcine skin tissue (2 cm × 2 cm) on the surface of a glass support thermostated at 32.0 ± 0.5 °C. An incision of 2 cm was made by a scalpel on the skin sample in order to simulate a wound. The latter was filled with 500 μL of SWF and the hydrogel film was put in contact with the simulated wound by applying a light force (0.5 N) for 60 sec. After this time, the force was removed and the hydrogel film kept in contact with the skin for further 60 sec and then put in traction. The force necessary for film detachment to skin was measured and expressed as average of three measurements (*n* = 3).

#### 3.5.5. In Vitro Release of Extract from The Hydrogel Film

OLE In vitro release from the hydrogel film was evaluated by vertical Franz diffusion cell (USP <1724>; PermeGear, Inc., Bethlehem, PA, USA, diameter 20 mm). A cellulose membrane (Whatman 41, Whatman GmbH, Dassel, Germany) was placed between the two chambers. The receptor chamber was filled with SWF as receptor medium (15 mL), thermostated at 32.0 °C ± 0.5 and magnetically stirred (600 rpm). The hydrogel film (circle of 3.14 cm^2^) was placed on top of the cellulose membrane. The donor phase was represented by 2 mL of a 0.025-N K_2_CO_3_ solution (simulating air CO_2_) [43]. All openings including donor top and receptor arm were occluded with parafilm^®^ to prevent solvent evaporation. At regular time intervals (5, 10, 15, 30, 45, 60, 120, 240, 300, 360, 420, 480, 1440 and 2880 min) samples of the receiving phase were withdrawn and OLE content was measured by a UV-vis spectrophotometer (UV-Visible Agilent model 8453, Agilent Technologies, Cernusco sul Naviglio MI, Italy) accomplished using a standard curve in SWF (λ_max_ = 280.0 nm, r = 0.9998). All experiments were performed in triplicate, each result represents an average of three measurements and the error was expressed as standard deviation (±SD).

## 4. Conclusions

The manuscript described the use of the non-edible outside layers of the onion cultivar Rojo Duro for the preparation of a hydrogel film for dermal application. This study proved that medical devices could be developed by recycling a waste material from agricultural and food processing industries. The use of the non-edible outside layers as a source of active ingredients is an efficient and smart approach for avoiding the expensive disposal procedures while producing a high-value added product.

The extract (OLE) deriving from the non-edible outside layers was obtained by using eco-friendly solvents thus without the production of further waste. OLE showed interesting antioxidant, radical scavenging, anti-inflammatory and antibacterial activities. As these properties were suitable for wounds treatment, OLE was formulated as autoadhesive biocompatible hydrogel film for application on injured skin with the aim to promote the healing process through synergic mechanisms. This could represent an effective alternative to conventional antibacterial therapies, limiting the use of antibiotics and thus the resistance problem.

OLE (10.92 mg/cm^2^) was released from the hydrogel film by a sustained release reaching effective concentrations to (i) exert the antibacterial activity against *S. epidermidis, S. aureus* and *E. faecalis* (common strains responsible for wounds infections); (ii) obtain the anti-inflammatory activity and at the same time and iii) maintain cells viability.

The hydrogel film is biocompatible, its composition is simple (PVP K90, NaCMC, bentonite), allows a rapid adhesion to skin, without the use of adhesives, a mechanical protection of the wound and an easy/pain-free removal by washing. Moreover, the hydrogel film is self-applicable. The last property, together to that described above, fulfills the compliance of the patient. The hydrogel film is green, scalable and cheap, thus resulting advantageous for the industry.

The obtained results suggest that the developed formulation represents an innovative and effective strategy useful for wounds treatment.

## Figures and Tables

**Table 1:** Recovery extract yield.

Extraction Conditions: Solvent, Temperature	Recovery Yield (%) ^a^
abs EtOH, RT ^b^	6.4
70% EtOH, RT ^b^	8.7
abs EtOH, 60 °C	7.9
70% EtOH, 60 °C	9.7

^a^ mean of two experiments performed in duplicate. ^b^ RT room temperature.

**Table 2:** Minimum inhibitory (MIC) and minimum bactericidal (MBC) values of OLE and the reference antibiotic ampicillin expressed as mg/mL ± SD (*n* = 3).

	*S. epidermidis*	*S. aureus*	*L. innocua*	*E. faecalis*
OLE MIC	0.47 ± 0.00	0.94 ± 0.00	3.75 ± 0.00	3.75 ± 0.00
OLE MBC	0.94 ± 0.00	1.88 ± 0.00	7.50 ± 0.00	7.50 ± 0.00
Ampicillin MIC	0.13 ± 0.00	0.13 ± 0.00	0.50 ± 0.00	0.50 ± 0.00
Ampicillin MBC	0.50 ± 0.00	0.25 ± 0.00	1.00 ± 0.00	4.00 ± 0.00

**Table 3:** Inhibition halos measured for the hydrogel films B1–B3. Results are expressed as mm ± SD (*n* = 3, n.i. = no inhibition).

Hydrogel Film (OLE mg)	*S. epidermidis*	*S. aureus*	*L. innocua*	*E. faecalis*
B1 (3.64)	23.00 ± 0.00	16.33 ± 0.58	n.i.	n.i.
B2 (10.92)	28.67 ± 0.58	20.33 ± 0.58	21.67 ± 0.58	21.00 ± 0.00
B3 (18.21)	25.67 ± 0.58	21.00 ± 0.00	21.67 ± 0.58	21.33 ± 0.58

n.i. no inhibition.

## Supplementary Materials

The following are available online at https://www.mdpi.com/1420-3049/25/2/318/s1, Figure S1: The TGA curve shows three steps: the first one until 200 °C corresponding to the vaporization of residual water (13.6%), the second step until 500 °C due to the decomposition of the organic components, the third one above 500 °C characterized by a mineral residue (22.78%) due to bentonite content. Figure S2. PVP K90 shows a broad endothermic peak between 45 and 120 °C due to the evaporation of bound water or moisture. For bentonite it is detectable a broad band between 60 and 100 °C attributable to adsorbed water loss. NaCMC shows a glass transition temperature (Tg) at 75 °C, corresponding and an exothermic peak at 284 °C due to the melting and the crystallization transition. Pre OLE thermogram shows that thermal process occurs at 80 °C probably related to the loss of volatile constituent of the sample (ethanol). No thermal decomposition was observed in the temperature range investigated. For the hydrogel film B2 a glass transition temperature (Tg) at 228 °C was observed. The spectra are shown in arbitrary units (a.u.) and shifted for clarity. Figure S3. Swelling % and erosion % of the hydrogel film B2 in SWF thermostated at 32 °C for 480 min. Table S1. Hydrogels compositions. Table S2. Compositions of hydrogels loaded with different OLE percentages. Table S3. Bacterial strains and growth conditions.
