# Supplementary material for: Bioadhesive Polymeric Films Based on Red Onion Skins Extract for Wound Treatment: An Innovative and Eco-Friendly Formulation
Source: Molecules. 2020 Jan 13;25(2):318. doi: 10.3390/molecules25020318 (PMC7024171; doi:10.3390/molecules25020318)
Supplement: Supplementary file 1 [file molecules-25-00318-s001.pdf]

Supplementary material

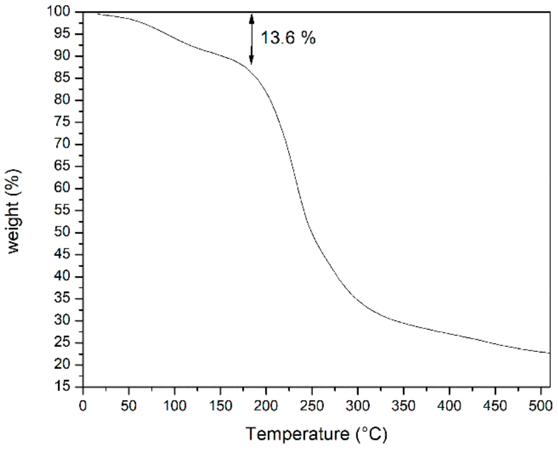

Figure S1. The TGA curve shows three steps: the first one until ~ 200°C corresponding to the vaporization of residual water (13.6%), the second step until ~ 500°C due to the decomposition of the organic components, the third one above 500°C characterized by a mineral residue (~ 22.78%) due to bentonite content.

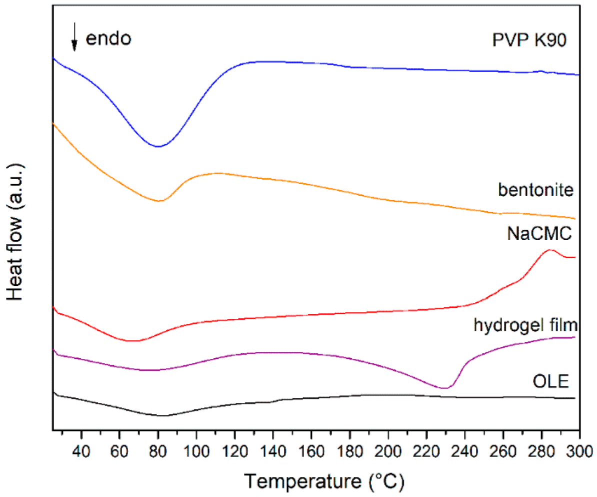

Figure S2. PVP K90 shows a broad endothermic peak between ~ 45-120°C due to the evaporation of bound water or moisture. For bentonite it is detectable a broad band between ~ 60-100°C attributable to adsorbed water loss. NaCMC shows a glass transition temperature (Tg) at ~ 75°C, corresponding and an exothermic peak at ~ 284°C due to the melting and the crystallization transition. Pre OLE thermogram shows that thermal process occurs at ~ 80°C probably related to the loss of volatile constituent of the sample (ethanol). No thermal decomposition was observed in the temperature range investigated. For the

hydrogel film B2 a glass transition temperature (Tg) at 228°C was observed. The spectra are shown in arbitrary units (a.u.) and shifted for clarity.

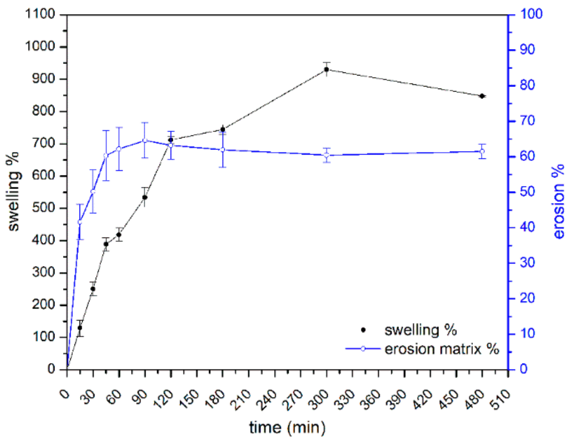

Figure S3. Swelling % and erosion % of the hydrogel film B2 in SWF thermostated at 32°C for 480 min.

Table S1. Hydrogels compositions.

Hydrogel	NaCMC (w/w %)	PVP K90 (w/w %)	bentonite (w/w %)	glicerol (w/w %)	ultrapure water (w/w %)
**1**	2.0	0.1	1.0	10.0	86.9
**2**	2.0	0.1	2.0	10.0	85.9
**3**	2.0	0.1	3.0	10.0	84.9
**4**	2.0	0.1	4.0	10.0	83.9
**5**	3.0	0.1	4.0	10.0	82.9
**6**	3.0	0.1	1.0	10.0	85.9

Table S2. Compositions of hydrogels loaded with different OLE percentages.

Hydrogel	OLE (w/w %)	NaCMC (w/w %)	PVP K90 (w/w %)	bentonite (w/w %)	glicerol (w/w %)	ultrapure water (w/w %)	*mg OLE/cm^2
**B1**	1.0	3.0	0.1	1.0	10.0	84.9	3.64
**B2**	3.0	3.0	0.1	1.0	10.0	82.9	10.92
**B3**	5.0	3.0	0.1	1.0	10.0	80.9	18.21

*amount of OLE/cm^2 in the hydrogel films after drying in oven.

Table S3. Bacterial strains and growth conditions.

**Gram positive bacteria**	**Growth conditions**
Staphylococcus epidermidis WDCM 00036	37°C for 24 ± 2 h
Enterococcus faecalis WDCM 00087	37°C for 24 ± 2 h
Lactobacillus sakei WDCM 00015	30°C for 48-72 h
Bacillus cereus WDCM 00001	30°C for 24 ± 2 h
Listeria innocua WDCM 00017	37°C for 24-48 h
Clostridium perfrigens WDCM 00007	37° C for 24-48 h anaerobic atmosphere
Staphylococcus aureus WDCM 00034	37°C for 24 ± 2 h
**Gram negative bacteria**	
Pseudomonas aeruginosa WDCM 00025	25°C for 24-48 h
Klebsiella pneumoniae WDCM 00097	37°C for 24 ± 2 h
Enterobacter aerogenes WDCM 00175	37°C for 24-48 h
Escherichia coli WDCM 00013	37°C for 24 ± 2 h
